# Sclerectomies in nanophthalmos and idiopathic uveal effusion syndrome: a systematic review

**DOI:** 10.1007/s00417-025-06908-4

**Published:** 2025-07-15

**Authors:** Leonor Braga de Sousa, João Barbosa-Breda

**Affiliations:** 1 Faculty of Medicine of the University of Porto, Porto, Portugal; 2 RISE-Health, Department of Surgery and Physiology, Faculty of Medicine of the University of Porto, Porto, Portugal; 3 Department of Opththalmology, Centro Hospitalar E Universitário São João, Porto, Portugal; 4 Research Group Ophthalmology, Department of Neurosciences, KULeuven, Louvain, Belgium

**Keywords:** Nanophthalmia, Nanophthalmos, Uveal effusion syndrome, Idiopathic uveal effusion syndrome, Scleral surgery

## Abstract

**Purpose:**

Different scleral decompression surgical procedures have been proposed for the treatment of idiopathic and nanophthalmic uveal effusion syndrome (UES). The aim of this review is to describe the different surgical approaches reported in the literature and compare the outcomes and complications between them.

**Methods:**

We searched PubMed/MEDLINE, Scopus and Web of Science for all articles that reported scleral decompressive surgical procedures for idiopathic and/or nanophthalmic UES treatment, as well as publications reporting prophylactic surgeries for uveal effusion in nanophthalmic eyes. Risk of bias was assessed using the Cochrane proposed tool for randomized controlled trials– RoB2 and the JBI checklist for case series and for cohort studies.

**Results:**

Twenty-eight articles were included and reviewed. Sclerectomies were the most frequently reported procedures, associated or not with sclerostomies or sclerotomies. Following in frequency were sclerostomies and sclerotomies alone, whereas vortex vein decompression (VVD) was the less frequently reported surgery. Overall, the articles demonstrated positive results in the resolution of uveal effusion and retinal/choroidal detachment, as well as in the prevention of uveal effusion in nanophthalmic eyes. Improvement in visual acuity (VA) was reported by most authors, except in cases with long-term retinal detachments (RD), where retinal damage prevented an enhancement of VA even with good anatomical results. Moreover, three studies included the use of adjunctive treatment to the surgeries, particularly mitomycin C (MMC) and intravitreal anti-VEGF injections. Complications of scleral decompression surgeries were reported in only ten articles and the most frequent and serious ones included *phthisis bulbi*, retinal and suprachoroidal hemorrhage, and vortex vein incision, among others.

**Conclusion:**

In general, scleral decompressive surgeries showed efficacy in treating and preventing UES. However, bigger studies would be necessary to minimize possible bias and to draw more solid conclusions regarding the benefit of surgical management of these patients, compared to a conservative one, and to better understand if adjunctive treatment can be, in fact, beneficial or not.

**Key messages:**

***What is known***

- The best treatment for idiopathic and nanophthalmic uveal effusion syndrome is not yet clear among ophthalmologists, particularly due to their rare incidence. Many surgical techniques have been proposed for the management of these conditions, but a bigger study on this topic has not yet been made.

***What is new***

- We performed a systematic review of all published literature on surgical approaches for idiopathic and nanophthalmic uveal effusion syndrome, including its prophylaxis in nanophthalmic eyes.
- Most studies demonstrated good results with the use of scleral decompressive surgeries, particularly sclerectomies, sclerostomies and sclerotomies. Bigger studies with control groups are, however, necessary to create more robust evidence.

**Supplementary Information:**

The online version contains supplementary material available at 10.1007/s00417-025-06908-4.

## Introduction

UES is a very rare condition characterized by choroidal effusion and serous choroid/retinal detachment [1]. Its prevalence is not certain. Sharma et al. recorded an estimated incidence of 1.2 per 10 million population a year in the United Kingdom, but the authors mention this is likely an underestimation, given its frequent misdiagnosis [2]. Other terms have been used in the literature as synonyms of UES, such as choroidal effusion, ciliochoroidal effusion and choroidal/ciliochoroidal detachment [3]. UES should be a diagnosis of exclusion, after other possible pathologies have been excluded, such as uveal melanoma, metastases, Vogt-Koyanagi-Harada disease, choroiditis, posterior scleritis, central serous chorioretinopathy. It usually presents in cases of normal intraocular pressure (IOP) and in the absence of inflammation [3]. Uyama et al. described three different subtypes of UES, according to the size of the eye and scleral abnormalities. The type I is found in nanophthalmic eyes with short axial length (AL) and thick sclera, type II in normal sized eyes with abnormally thick sclera and type III in eyes with normal axial lengths and sclerae, usually called as idiopathic uveal effusion syndrome (IUES) [1, 4]. In this review we focused on nanophthalmic and idiopathic UES.

Nanophthalmos is a phenotypical subtype of microphthalmos, which is a developmental eye disorder characterized by small eyes, at least two standard deviations smaller than the average AL of normal eyes in the same age group [1]. Prevalence of microphthalmos in the population is not certain, however Day et al. estimated a prevalence between 0.002% and 0.017% in the European population [5]. Nanophthalmic eyes result from a growth arrest of the eye after the closure of the embryonic fissure and, therefore, present a globally small eyeball, unlike posterior microphthalmos that have a small posterior segment, but a normal-sized anterior segment [1, 6, 7]. In addition, nanophthalmic eyes have a normal eye structure without any congenital malformations but exhibit thickening of the choroid and sclera [3, 8]. Corneal diameter and lens are usually normal in these eyes, resulting in a high lens:eyeball ratio and shallow anterior chamber (AC), with predisposition for angle closure spectrum diseases [3]. In addition, these eyes usually present a short AL and marked hyperopia [3], which is usually the first symptom to manifest in childhood [1]. These patients often go undiagnosed for several years, before other complications, such as chronic angle closure glaucoma (CACG) and UES manifest [1, 9]. Some other reported clinical characteristics of these eyes include poor dilation of pupils, wide ocular pulse amplitude, narrow palpebral fissures and choroidal detachments [3, 10]. Most authors consider that the bigger risk of UES in these eyes derives from the increased scleral thickness, which leads to venous congestion and impaired vascular drainage of these eyes [3]. Disorganization of collagen fibers in the sclera of these patients has been reported and this manifests as a reduced scleral elasticity that compromises the outflow of proteins and glycosaminoglycans from these eyes as well as the vortex vein drainage [3, 8, 11]. Furthermore, these eyes present greater risk of complications during and after cataract, glaucoma and retinal surgeries, such as worsening of angle closure glaucoma, suprachoroidal hemorrhage, UES, among others [3, 8]. Moreover, anatomical features such as a small ocular volume, crowded AC, deeply set eye in the orbit and narrow palpebral fissures, reduce surgical access and make almost all surgical procedures more challenging [3]. UES can occur spontaneously or as a surgical complication, probably due to postoperative inflammation that leads to an increased protein leakage from the vessels and to some possible hypotony during the procedures that reduces transscleral hydrostatic pressure [10].

Both IUES and nanophthalmic UES share very similar characteristics and should be a diagnosis of exclusion [3]. IUES occurs mostly in middle aged men, whereas nanophthalmic UES occurs in both sexes and usually affects both eyes [11]. This disorder shows a relapsing course and around half of the patients that undergo surgery, eventually need surgery in the other eye [3]. In IUES, aging and hormonal changes can alter scleral permeability to proteins and result in vortex vein compression, that would explain the middle-age manifestation of the disorder in these eyes [11].

**Fig. 1:**
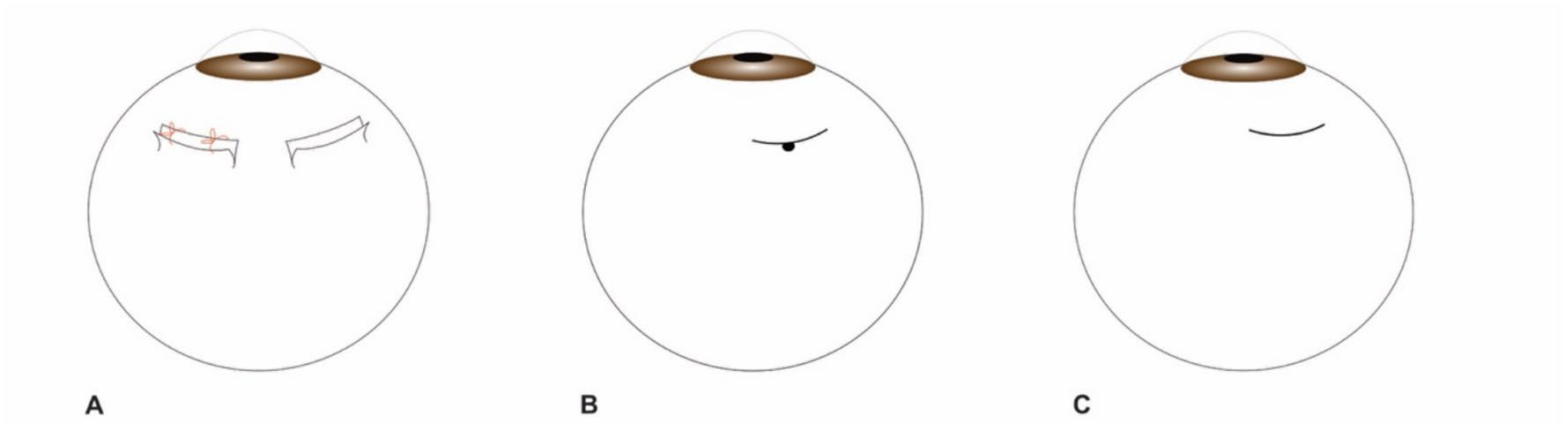
Sclerectomies (**A**); sclerostomy (**B**); sclerotomy (**C**)

Treatment of UES, both in nanophthalmic eyes or in cases of IUES, is frequently surgical. Different authors have proposed different surgical approaches, but the best surgical treatment of these patients is still unclear. Vortex vein decompression (VVD) was one of the first suggested procedures [12], but over the years some authors have claimed it is a technically challenging procedure, that comprises many risks, namely vortex vein puncture [3, 13–15]. Over the years, it has been substituted by sclerectomies and/or sclerotomies [3, 11]. It is important to consider that different names are used for the procedures among the literature. In general, sclerectomies refer to the removal of a scleral flap (that can be totally removed or kept attached and, in this case, sutured or not), sclerostomies to the puncture of sclera with removal of a small piece of it and sclerotomies to a mere scleral incision, as demonstrated in Fig. 1. These techniques can be used concomitantly or not. VVD consists of a deroofing of the vortex veins where a sclerectomy is performed over the vortex veins’ route in the sclera. Non-surgical treatment of UES has been reported, especially the use of high-dose systemic corticosteroids, yet this treatment seems to have little efficacy [3]. Long-term use of NSAIDs in association with laser photocoagulation has been documented [16], as well as the topical use of NSAIDs alone [17]. Carbonic anhydrase inhibitors and local prostaglandin analogues have shown some utility in the absorption of subretinal fluid (SRF) in nanophthalmic UES [18] and anti-VEGF intraocular injections have also been reported by Guo et al. [19].

The central goal of this review is to describe the different surgical techniques reported in the literature for the treatment and prophylaxis of idiopathic and nanophthalmic UES and to compare the outcomes and complications between them.

## Methods

### Search methods

This review was conducted according to the Preferred Reporting Items for a Systematic Review and Meta-Analysis (PRISMA) guidelines.

A search of literature was performed in 3 electronic databases (PubMed/MEDLINE, Scopus and Web of Science) using the search queries shown in Online Resource 1. No search limits or filters were used and the last database search was made on September 8th 2024. Two additional relevant studies were obtained by citation tracking.

### Inclusion criteria

We included articles that comprehended patients with: (1) nanophthalmos/posterior microphthalmos who were submitted to decompressive scleral surgeries, such as sclerectomies and sclerotomies, for treatment or prophylaxis of UES; (2) idiopathic UES who underwent decompressive surgeries.

### Exclusion criteria

**Fig. 2:**
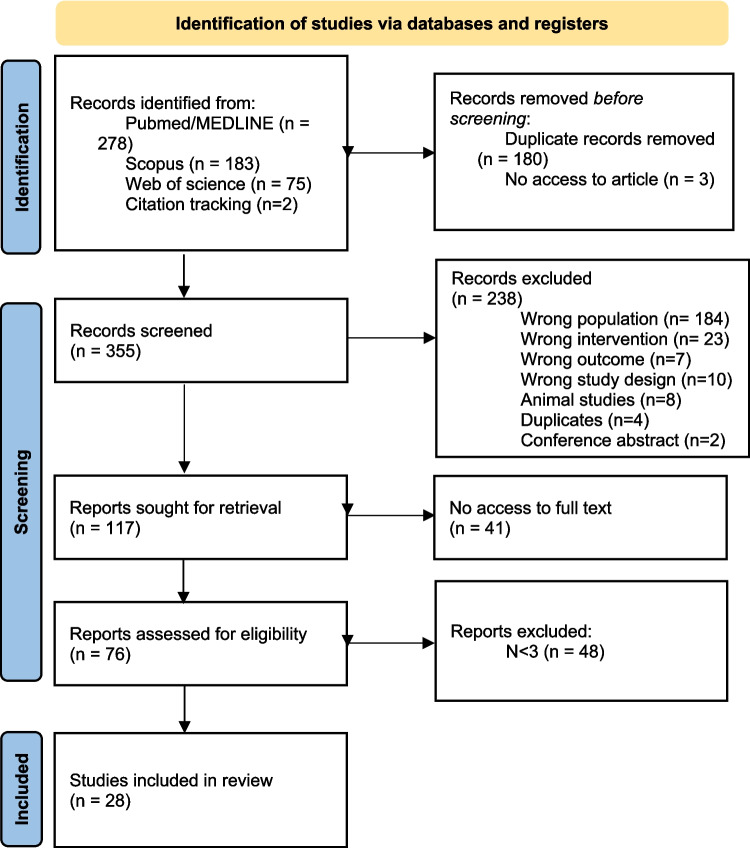
- PRISMA flowchart for study retrieval and selection

We excluded all studies comprised in at least one of the following exclusion criteria: animal studies, reviews, conference abstracts and studies with < 3 eyes. No language or year of publication restrictions were imposed. The exclusion criteria are summarized in Fig. 2.

### Outcomes evaluated

The main outcomes considered included: improvement of UES demonstrated as reattachment of the retina and/or choroid, improvement of VA during follow-up and main reported complications of the surgeries.

### Study selection and data collection

All articles retrieved from the databases were firstly screened based on the titles and abstracts and, in a second stage, based on the full text reading. Both steps were carried out by two independent researchers and, in case of disagreement, unanimity was reached through discussion.

Two independent researchers retrieved the following data from the articles: year of publication, country, study design, number of patients, number of eyes (considered those submitted to decompressive surgery); diagnosis of nanophthalmos/posterior microphthalmia and/or idiopathic UES, pre-operatory choroidal/retinal detachment, pre- and post-operatory VA (converted to logarithm of the Minimum Angle of Resolution (logMAR) when presented in Snellen scale, according to a previously published conversion formula [20]), conservative treatment (if made prior to surgery), type of surgery, adjunctive treatment for surgery (if used), surgical complications and post-operatory changes in choroidal/retinal detachments. In addition, three authors were contacted for details about the performed surgical procedures, but we received only one answer.

Given that the terms “uveal effusion syndrome” and “choroidal detachment/effusion” are usually used interchangeably, we considered all cases where it was only mentioned UES as having choroidal detachment and vice-versa. In addition, the serous RD, when present in these patients, are a result of the choroidal effusion and, for this reason, we opted to report choroidal/retinal detachment together. When only a reattachment of the retina was mentioned in the results of the studies, we considered it as an improvement of the UES. Different authors use different criteria for the diagnosis of nanophthalmos, but most of them use more than one parameter for the diagnosis (short AL, usually < 21 mm, shallow anterior chamber, hyperopia, retinal-choroidal-scleral thickness > 1.7 mm, among others). We considered the diagnosis of nanophthalmos in every patient indicated as such by the respective authors.

Some authors differentiate between “light perception” (LP) and “light projection” in the VA values. Given that all values presented in Snellen scale were converted to logMAR to enable the calculation of the mean value and that most authors simply classify all of these as LP, we decided not to make that distinction and considered all these cases as LP.

### Assessment of risk of bias in the included studies

All articles included in this review were assessed for potential risk of bias by an independent researcher (LBS), supervised by a senior researcher (JBB).

The randomized controlled trial (RCT) was assessed according to the Cochrane Handbook for Systematic Reviews [21]. We used the second version of the Cochrane proposed tool for RCTs– RoB2 [22], available as supplementary material (Online Resource 2.1). The risk of bias was considered medium, with some concerns regarding the measurements of the outcomes.

The case series and cohort studies were evaluated using the 2020 version of the JBI checklist for case series and for cohort studies, respectively [23, 24], and none of the studies was excluded due to a high risk of bias. The results are summarized in Online Resources 2.2 and 2.3.

## Results

Twenty-eight studies were included in our review after removing duplicates and performing title and abstract screening, as well as full-text screening. Since it is a rare condition, only a few large studies have been made on this theme and most of the articles included were retrospective chart reviews and case series. Only one RCT on this topic was retrieved. The results of each study are summarized in Table 1.

**Table 1:** Summary of the studies

Author and year	Study design	No. eyes	Nanophthalmos(% of eyes)	UES pre-op (% of eyes)^a^	RD pre-op (% of eyes)	VA pre-op (mean logMAR)	Conservative treatment	Surgery	UES resolution/development (% of eyes)	RD resolution/development (% of eyes)	VA postop (mean logMAR)
Brockhurst [12]	Case series	10	Yes	100%	100%	2,33	Steroid therapy	VVD (6 × 4 mm almost FTS anterior to the point of veins’ exit from sclera + meridional sclerotomies in each bed of dissection close to the horizontal meridian + sclerotomies to expose, and if needed perforate, the choroid)	80%	80%	1,97
Brockhurst [25]	Case series	4	Yes	25%	25%	2,03	NA	VVD + lamellar resection in 4 quadrants (+ cataract surgery in 3 eyes; + sclerotomies in 1 eye; + SRF drainage in 1 eye)	0	0	1,08
Casswell [13]	Case series	6	0%	100%	100%	NA	NA	3-VVD; 4- PTS	100%	100% (2 partial; 4 total)	NA
Desai [26]^b^	Case series	6	66,7%	25%	100%	1,60	Steroid therapy	PTS + SRF drainage	100%	100%	1
Fan [27]	Retrospective cohort	19	100%	0	0	1,30	NA	Phacoemulsification + anterior vitrectomy + 3 × 2 mm PTS 7-8 mm posterior to the corneoscleral limbus + 2 mm sclerotomy	0	0	1,10
Faulborn [28]	Case series	5	75%	100%	100%	1,50	High-dose systemic steroids	8 × 2 mm FTS 4 mm from limbus	100%	100%	0,48
Ghazi [29]	Case series	6	0	100% (choroidal swelling)	50%	0,54	NA	4 mm full-thickness incision + scleral punch OR 4 × 4 mm PTS + full-thickness incision + scleral punch	100%	100% (2 total; 1 partial)	0,35
Guo [19]	Case series	3	33%	100%	100%	0,62	NA	5–7 × 8 mm PTS in 4 quadrants + intra-vitreal VEGF injections	100%	100%	0,66
Jin and Anderson [10]	Case series	15	100%	27%	NA	NA	NA	V-shape FTS (if UE) OR sclerectomies/sclerotomies + cataract/glaucoma surgery	100%/100%	100%/100%	NA
Johnson and Gass [30]	Case series	23	0	100%	87%	0,70	High-dose systemic or periocular CCT	5 × 7 mm PTS + 2 mm sclerotomy + 1-2 mm scleral punch in all quadrants (19 eyes) (1 eye- 2 quadrants; 1 eye- 3 quadrants; 1 eye- 6 PTS without sclerotomies/sclerostomies; 1 eye- triangular loosely sutured FTS + sclerotomy)	96%^c^	83%	0,48
Kong [31]	Case series	5	50%	100%	100%	0,17	Oral prednisolone (in 40%)	5–6 × 2-3 mm FTS loosely sutured flap 6-8 mm from limbus in 4 quadrants (5 FTS in 3 quadrants (1 eye); + SRF drainage (1 eye)	100%	100%	0,25
Liu [32]	Case series	3^b^	100%	100%	100%	1,33	NA	Lamelar sclerotomies	100%	100%	NA
Maggio [33]	Case series	5	Type I (40%); type II (40%); type III (20%)	40%	100%	1,48	Oral CCT (+ IV CCT in 1 eye; + IV CCT + azathioprine in 1 eye; + retrobulbar CCT in 1 eye)	4 × 4 mm PTS in inferior quadrants (+ phacoemulsification in 1; + SRF drainage in 2; + phacoemulsification + 2 FTS in 1)	100%	100%	0,74
Mansour [34]	Case series	5	100%	100%	100% (lens touch)	2,70	Oral CCT (80%)	PTS in 4 quadrants (+ SRF drainage in 60%)	100%	100%	0,70
Mansour [35]	Case series	8	100%	100%	0	0,69	NA	PTS in all quadrants (except ¼ of superotemporal quadrant) from rectus muscle insertion past the vortex veins (canthotomy in 80%)	100%	NA	0,51
Özdek [36]	Case series	14	100%	100%	100%	1,5	NA	6 × 4 mm PTS + sclerotomy in 4 quadrants (only 2 quadrants in 3 eyes w/glaucoma) (+ SRF drainage in 1 eye) (2 eyes recurred and had 2nd surgery w/MMC)	100% (total in 11; partial in 1; recurrence in 2)	100% (total in 11; partial in 1; recurrence in 2)	0,92
Ozgonul [14]	Case series	6	83% (2 in the spectrum, borderline values)	33%	33%	0,41 (the values of 2 eyes weren’t reported)	Oral CCT in 50% (+ topical dorzolamide + steroid-sparing agents in 1 eye)	4 × 4 mm PTS 1 mm posterior to rectus insertions in 3 or 4 quadrants (+ cataract surgery in 4 eyes; + MMC + punch sclerostomy + SRF drainage in 1 w/recurrence; + MMC in 1 recurrence; + VVD in 2nd surgery of 1 eye w/o improvement)	100%/100%	100%/100%	0,39 (the values of 2 eyes weren’t reported)
Rajendrababu [37]	RCT	60	100%	0	NA	1,34	NA	Phacoemulsification/SICS (+ 4 × 4 mm sutured PTS + 2 × 2 mm sclerostomy 10-12 mm posterior to limbus in 48% of eyes)	6,7% (didn’t undergo PTS + sclerostomy)	1,6% (underwent PTS + sclerostomy)	0,69
Rajendrababu [38]	Retrospective chart review	232	100%	10,77%	NA	0,78 (median)	NA	SICS/phacoemulsification (+ 3-4 mm superficial scleral flap + 2 mm full-thickness cut to expose the choroid in the inferotemporal quadrant in 22%)	NA	NA	0,48 (median)
Shah [39]	Case series	3	0%	100%	100%	0,23	NA	PTS in all quadrants + 2 inferior incisional sclerotomies + suprachoroidal fluid drainage	100%	100%	0,1
Sharma [2]	Surveillance study	11	NA	100%	NA	0,48 (median)	Systemic acetazolamide + bevacizumab injections in 9%	FTS (+ MMC in 9%)	Total 27,3%; partial 45,5%	NA	0,55 (median)
Shen [40]	Case series	33	0%	100%	100%	NA	NA	5 × 7 mm PTS + 1 × 2 mm full-thickness sclerostomy in all quadrants	100%	100%	Increased in 91%; unchanged in 9%
Uyama [4]	Case series	19	31,6%	100%	84,2%	1,17	NA	4 × 5 mm loosely sutured PTS + 3 × 4 mm FTS in inferior quadrants (+ all quadrants in 10,5%; + FTS w/flap removal in 5,3%; + additional surgeries in 42,1%)	94,1% in types I and II UES; 0% in type III	94,1% in types I and II UES; 0% in type III	0,93
Weng [41]	Case series	10	0%	100%	100%	0,55	NA	5–7 × 7-10 mm PTS + 1–1,5 mm scleral punch	100%	100%	0,10
Wu [40]	Case series	4	100%	0%	0%	1,83	NA	Cataract surgery + 2 or 4 quadrant lamellar scleral resections	0%	0%	1,60
Yalvac [42]	Case series	20	100%	0%	0%	0,24	NA	Trabeculectomies (+ MMC) + FTS 3 mm posterior to limbus in inferior quadrants	50% (40% transient; 10% required drainage)	NA	0,18
Yepez [43]	Case series	3	0%	100%	100%	1,10	Oral prednisolone	Oblique sclerotomy 13 mm posterior to limbus w/EX-PRESS shunt in quadrant of maximal choroidal effusion	100%	100%	0,26
Zhou [44]	Retrospective cohort	106	58,5%	100%	100%	< 1 in 73,6%; [0,70–1] in 26,4%	Periocular corticosteroids in 29%	3–4 × 5-6 mm PTS + 2 × 2 mm sclerostomies in inferior quadrants	100%	100%	< 1 in 10,4%; [1–0,82] in 64,1%; 0,60 in 25,5%

*UES* uveal effusion syndrome, *RD* retinal detachment, *VA* visual acuity, *PTS* partial thickness sclerectomy, *FTS* full thickness sclerectomy, *VVD* vortex vein decompression, *SRF* subretinal fluid, *CCT* corticosteroids, *MMC* mitomycin C, *SICS* small incision cataract surgery, *NA* not applicable

^a^Choroidal detachment was considered as UES

^b^Only the patients that underwent scleral surgery was described in this table

^c^In 3 eyes a second surgery was necessary: reopening of sclerotomies, removal of scar tissue from sclerectomies as well as periocular corticosteroid injections in 2 plus partial SRF drainage in 1 of these; addition of 2 superior sclerectomies and sclerostomies in 1

In most articles, patients were submitted to sclerectomies with different characteristics and details between them, such as size and depth.

Casswell et al. [13] described six eyes with IUES and RD in non-nanophthalmic patients. Four eyes underwent near full-thickness sclerectomies, resulting in total or partial RD resolution, although one eye relapsed. One eye had undergone VVD without success. A very similar technique was performed by Mansour et al. [35] that reported eight nanophthalmic eyes treated with 90% thickness sclerectomies in more than 260º of sclera, observing an improvement in logMAR from 0.69 to 0.51 and complete choroidal reattachment. Maggio et al. [33] reported five eyes initially treated unsuccessfully with oral corticosteroids (CCT) (one eye was also treated with azathioprine and intravitreal CCT, another with retrobulbar CCT and two eyes with intravenous (IV) CCT) and later undergoing 2/3 thickness sclerectomies, all achieving RD resolution, though one case recurred and required additional surgery. Improvement in VA was seen in all but one eye. Liu et al. [32] described three nanophthalmic eyes treated with lamellar sclerectomies, all showing retinal reattachment at the last follow-up. Faulborn and Kölli [28] reported five eyes (four nanophthalmic) with UES and RD that underwent full-thickness 8 × 2 mm sclerectomies in all quadrants. All but one eye had done IV corticosteroid treatment without success prior to surgery. All eyes had RD resolution and improved VA (1.50 to 0.48 logMAR) after surgery. Kong et al. [31] studied five eyes with UES and RD, three of which were nanophthalmic. The nanophthalmic patients underwent full-thickness scleral flaps in all quadrants (5–6 × 2-3 mm) with loose suturing. The two non-nanophthalmic patients received oral corticosteroids without success before undergoing the same surgery. One of them required additional sclerotomies and the other required SRF drainage. Postoperatively, all eyes showed RD resolution, and mean VA changed from 0.21 to 0.31 logMAR as it improved in only two of the eyes.

In some other series, sclerectomies were associated with SRF drainage. Mansour et al. [34] analyzed five nanophthalmic eyes with lens-touch RD, performing similar sclerectomies in all quadrants and SRF drainage in 60% of cases, with logMAR improving from 2.70 to 0.70. Desai et al. [26] evaluated 25 eyes, 15 of which with nanophthalmos and UES, observing better outcomes in the group that underwent partial thickness sclerectomies with SRF drainage (100% RD resolution and improvement in VA) compared to conservative treatment with corticosteroids (83% RD resolution).

Among the articles retrieved, many documented patients submitted to partial thickness sclerectomies associated with sclerotomies. Shah et al. [39] reported three eyes from two patients with IUES treated with 90% thickness sclerectomies in all quadrants and two inferior sclerotomies with balanced salt solution (BSS) injection. All eyes showed resolution of the detachment, and VA improved in two eyes (from 0.30 to 0.10 logMAR). Özdek et al. [36] described 14 nanophthalmic eyes with UES and RD that underwent 2/3 to ¾ thickness sclerectomy flaps, with flap removal and a central linear sclerotomy. The retina reattached in 11 eyes and partially in 1, with recurrence in 2. The mean VA improved from 1.50 to 0.92 logMAR. A severe case with retina-lens touch underwent SRF drainage and showed unexpectedly good results. Sharma et al. [2] reported 11 eyes with UES that underwent a similar technique of partial thickness sclerectomies with a central sclerotomy which demonstrated partial improvement in five patients, total resolution in three and zero response in the other three. Jin and Anderson [10] published a case series with 4 nanophthalmic eyes with choroidal effusion that underwent successful unsutured full-thickness V-shaped sclerotomies. The authors believe that an unsutured drainage incision is sufficient and that scleral resections are not needed to obtain good results. Yepez and Arevalo [43] described a different technique using an oblique sclerotomy with a 25-gauge needle in the quadrant with the most fluid accumulation (determined by ultrasound). A P-50 EX-PRESS shunt was then inserted to facilitate drainage, with BSS injected into the AC when needed. This procedure was performed on three eyes with type 2 UES and RD, resulting in total retinal and choroidal reattachments and an improvement in mean VA from 1.10 to 0.26 logMAR. The authors described the technique as simple and easy to perform but acknowledged the need for long-term follow-up to assess its durability.

Another group of studies included sclerectomies combined with sclerostomies, including scleral punch techniques. Weng et al. [41] described 10 eyes with IUES and RD that underwent quadrant sclerectomies (5 × 7 mm to 7 × 10 mm, 1/2 to 2/3 thickness) with a 1–1.5 mm central scleral punch. All retinas reattached, and VA improved (0.55 to 0.10 logMAR), except for two long-standing cases, which showed minimal visual improvement. Johnson and Gass [30] reported 23 eyes with UES, 20 of which with RD, treated with half to 2/3 thickness sclerectomies, combined with sclerotomies and scleral punches, except for one eye who underwent 6 sclerectomies without scleral punches and another eye who underwent full thickness scleral flaps with sclerotomies. After six months, 96% of cases had UES and RD resolution, with a recurrence rate of 23%, but resolved spontaneously or after reoperation in every patient. Median VA improved from 0.70 to 0.48 logMAR. Ghazi et al. [29] performed a similar procedure using ultrasound guidance in six eyes with UES. Three had RD, two of which fully reattached, while one had partial reattachment. Mean VA improved from 0.54 to 0.35 logMAR. Uyama et al. [4] studied 19 eyes with UES types 1, 2 and 3, which underwent 2/3 thickness 4 × 5 mm loosely sutured scleral flaps with 3 × 4 mm full-thickness sclerectomies in the scleral beds of the inferior quadrants. VA improved from 0.97 to 0.72 logMAR (type 1) and from 0.93 to 0.66 logMAR (type 2). Retinal and choroidal reattachments were observed in all eyes with types 1 and 2, except for one eye with type 2 UES. However, eyes with type 3 UES showed no improvement neither in VA, nor in choroidal/retinal reattachment. Their findings support that UES types 1 and 2 respond well to surgery, whereas type 3 does not. Shen et al. [45] analyzed 33 eyes with UES and RD that underwent 4-quadrant lamellar sclerectomies (5 × 7 mm, 2/3 thickness) with full-thickness sclerostomy. VA improved in 30 eyes, while 3 had recurrent RD and required reoperation with removal of scar tissue, which led to successful resolution. Zhou et al. [44] performed a similar technique in 106 eyes (62 with type 1 UES and 44 with type 2 UES). After surgery, 96.2% of cases were successful with one procedure and 100% with two. Type 1 UES group had late recurrences in 7 eyes, but 3 reattached spontaneously. VA improved significantly in both groups. The authors considered that VA improvement was more influenced by the short duration of detachment than by the percentage of reattachment postoperatively.

Regarding UES prophylaxis rather than treatment, Ozgonul et al. [14] and Wu et al. [40] studied partial-thickness sclerectomies as a prophylactic approach for UES in nanophthalmic eyes before cataract surgery, with overall success. Ozgonul et al. [14] reported two nanophthalmic eyes who underwent 2/3 thickness prophylactic sclerectomies in 3 quadrants. One patient didn’t develop UES during the 3 years of follow-up, the other, however, developed UES and RD and was submitted to a revision of the sclerectomies plus a fourth sclerectomy, that resolved both the choroidal and the RD. Wu et al. [40] also reported four nanophthalmic eyes of 3 patients that had scleral surgery at the same time (1 eye) or prior to cataract surgery as UES prophylaxis (3 eyes). Two or four quadrant lamellar scleral resections were performed and none of the eyes developed choroidal serous effusions during follow-up. Fan et al. [27] compared three surgical approaches in 40 patients with nanophthalmos and angle-closure glaucoma. Triple surgery (prophylactic sclerectomies with sclerotomies, phacoemulsification, and anterior vitrectomy) resulted in better VA outcomes (*p* = 0.04) and lower RD and UES incidence than double (phacoemulsification and vitrectomy) or single glaucoma surgery (trabeculectomies or aqueous drainage device implantation). The sclerectomy group had fewer complications (*p* = 0.046). Jin and Anderson [10] also reported 9 eyes that underwent cataract surgery with prophylactic sclerectomies or sclerotomies and other 2 eyes that underwent glaucoma surgery with sclerectomies. None of these eyes developed UES during follow-up. Moreover, Yalvac et al. [42] reported a case series of 20 nanophthalmic eyes undergoing glaucoma surgery (trabeculectomy with MMC) with prophylactic full-thickness sclerotomies in the inferior quadrants over the pars plana 3 mm posterior to the limbus. Postoperatively, 10 eyes developed choroidal detachment, most of them transient, with only two requiring drainage. VA decreased in 65% of cases, improved in 10%, and remained stable in 25%, with choroidal detachment and retinal folds being the main causes of vision loss. Furthermore, Rajendrababu et al. [38] conducted a retrospective chart review of 114 nanophthalmic eyes, 51 of which underwent prophylactic sclerostomies during cataract surgery (12 with small incision cataract surgery (SICS) and 39 with phacoemulsification), while 63 served as a control group and had cataract surgery alone (20 SICS and 43 phacoemulsifications). Sclerostomies were performed in the inferotemporal quadrant by creating a 3-4 mm superficial scleral flap and making a 2 mm full-thickness cut to expose the choroid. A spatula was used to clear the suprachoroidal space, and the flap was sutured with cauterization to maintain the ostium open. The study found a significantly lower incidence of UES in the sclerostomy group (*n* = 4) compared to controls (*n* = 14) (*p* = 0.04) and a reduction in overall surgical complications (*n* = 12 in the sclerostomy group and *n* = 29 in the control group) (*p* = 0.08).

Rajendrababu et al. [37] also conducted the only RCT retrieved in this review, comparing cataract surgery with or without sclerectomies in nanophthalmic eyes. The study included 31 eyes in the control group (phacoemulsification or SICS) and 29 eyes in the sclerectomy group (same cataract surgery plus partial thickness sclerectomies in the inferonasal quadrants). The authors noted that sclerectomies facilitated cataract surgery by deepening the anterior chamber. Six months postoperatively, median VA improved significantly within both groups (control: 1.18 to 0.60 logMAR; sclerectomy: 1.30 to 0.78 logMAR), but the difference between groups was not statistically significant. Sclerectomy reduced surgical complications by 80%, though only the lower incidence of uveal effusion in this group reached statistical significance (*p* = 0.04). Postoperative ocular inflammation was higher in the sclerectomy group. Study limitations included non-blinded assessment of surgical complications and the use of two different cataract techniques, with SICS showing more postoperative complications. Overall, sclerectomy combined with cataract surgery appeared to reduce complications in nanophthalmic eyes.

### Studies mentioning vortex vein decompression

The term VVD is many times used interchangeably with sclerectomies. VVD was first described by Brockhurst [12] in 10 nanophthalmic eyes with UES and RD. The procedure involved scleral incisions to expose the intrascleral course of the vortex veins (deroofing) and sclerotomies to perforate the choroid. Eight eyes achieved total retinal reattachment, with mean VA improving from 2.33 to 1.71 logMAR. Better outcomes were observed in cases with shorter-term RD, reinforcing the importance of early treatment. In 1990, Brockhurst reported four additional cases, including one with UES and three undergoing prophylactic VVD with lamellar ressections during cataract surgery [25]. The eye with UES achieved full retinal reattachment, while the others had no post-operative detachments. Mean VA improved from 2.03 to 1.08 logMAR. The author suggested a two-month gap between VVD and cataract surgery to minimize inflammation before the second surgery.

Other studies also reported VVD, namely Casswell et al. [13] who performed VVD in three of seven eyes with UES, with two achieving full reattachment and one requiring additional partial thickness sclerectomies. Ozgonul et al. [14] documented a patient in the nanophthalmic spectrum with UES and RD who underwent VVD after failed sclerectomies, resulting in total reattachment and VA improvement from 0.60 to 0.30 logMAR. Both Casswell et al. and Ozgonul et al. noted VVD as a technically challenging procedure.

### Studies that used an adjunctive agent

Few studies mentioned adjunctive treatments for UES surgeries. Guo et al. [19] treated three eyes, one of which with nanophthalmos, with UES and RD using partial thickness 7 × 8 mm sclerectomies in all quadrants. After initial failure in two eyes and recurrence in the other, the same surgery was performed again in all eyes, this time associated with intra-vitreal anti-VEGF injections (ranibizumab and bevacizumab). RD resolved, but mean VA slightly declined from 0.62 to 0.66 logMAR.

MMC was used with decompressive surgeries in two studies. Özdek et al. [36] applied MMC in the second surgery of two eyes that recurred after sclerectomies with sclerotomies. Postoperatively, macular UE persisted in one eye, likely due to retinal pigment epithelium (RPE) damage, and the general reduction of SRF was less significant in this surgery compared to the first in both eyes. Ozgonul et al. [14] used MMC in revision surgeries for two eyes. One nanophthalmic eye that developed UE after cataract surgery and prophylactic partial thickness sclerectomies. Decompressive surgery was repeated three other times, using MMC in the second one, which did not improve success. In the third surgery punch sclerostomies and SRF drainage were performed and resolved the UE. The other eye had IUES and recurred 5 months after partial thickness sclerectomies in three quadrants, undergoing the same surgery a second time in all quadrants with MMC with good results.

### Complications

Complications from decompressive surgeries were reported in only a few studies. Brockhurst [12] and Casswell et al. [13] described phthisis bulbi and accidental vortex vein incision in two patients undergoing VVD. Intraocular lens dislocation after SRF drainage was reported by two authors [34, 36], with one also mentioning sectorial zonular dialysis [34]. Retinal hemorrhage occurred in one case of partial thickness sclerectomies [35].

In studies where decompressive surgery was performed prophylactically before cataract or glaucoma surgery, it was unclear whether the complications resulted from decompressive procedures or the main surgeries. Comparing control and sclerectomy/sclerostomy groups:

- Fan et al. [27] found fewer complications in patients undergoing triple surgery (phacoemulsification, anterior vitrectomy, and prophylactic sclerectomies) than in those having glaucoma surgery alone (trabeculectomies or aqueous drainage device implantation) (*p* = 0.046), and fewer follow-up procedures to treat complications were needed in this group (*p* = 0.001). Some of the reported complications in the triple surgery group included suprachoroidal hemorrhage and UE in approximately 5% of the eyes, in the double surgery group (phacoemulsification and anterior vitrectomy) suprachoroidal hemorrhage and vitreous hemorrhage in 7%, UE in 20% and RD in 13%. In the glaucoma surgery alone group 10% developed suprachoroidal hemorrhage and 20% UE, RD and aqueous misdirection, among other complications.
- Yalvac et al. [42] observed complications in patients undergoing trabeculectomy with full-thickness sclerectomies and MMC, but the authors noted difficulty in attributing them to either procedure. They observed choroidal detachment in 50% of eyes (10% of which needed drainage), late choroidal effusion in 25%, 40% of which with associated RD, and cataract formation in 35%.
- Rajendrababu et al. compared cataract surgery alone vs. with sclerostomies in two studies [37, 38]. In one of said studies, complications occurred in 29/63 eyes in the control group vs. 12/51 in the sclerostomy group, with a reduced risk in the latter (*p* = 0.08), though not statistically significant. In the control group they reported UES in 22%, RD in 2% and aqueous misdirection in 3%, whereas in the sclerostomy group UES was reported in 8%, RD in 2% and aqueous misdirection in 2%. In the other study (a RCT), choroidal effusions were observed in 13% of eyes and aqueous misdirection in 3% in the control group, and in the sclerostomy group RD and Descemet membrane stripping were reported in 3% of the eyes. The total number of complications were higher in the control group (*p* = 0.065).
- Wu et al. [40] described four nanophthalmic eyes undergoing cataract surgery with prophylactic scleral resections. Phthisis was reported in one eye postoperatively (25%).

Suprachoroidal hemorrhage was noted as a potential complication, especially in nanophthalmic eyes with high choroidal congestion during SRF drainage [14, 36]. Ozgonul et al. [14] performed SRF drainage associated with partial thickness sclerectomies and sclerostomies in one eye and Özdek et al. [36] performed SRF drainage in an eye with retina-lens touch. In both cases SRF drainage was considered safe. As mentioned above, Fan et al. [27] documented three cases of suprachoroidal hemorrhage, noting high pre-operative IOP as a risk factor. Additionally, Ghazi et al. [29] noted that choroidal swelling without true choroidal detachment can comprise a higher risk for suprachoroidal hemorrhage due to greater chances of choroidal penetration during scleral surgeries.

## Discussion

The results from the twenty-eight included articles in this review were overall positive and encourage the execution of decompressive scleral surgery, particularly sclerectomies and/or sclerostomies/sclerotomies either in patients with nanophthalmia as prophylaxis for UES, or as treatment of nanophthalmic and idiopathic UES. The single RCT included in this review demonstrated a statistically significant lower incidence of postoperative UE in nanophthalmic patients submitted to prophylactic sclerectomies in association with cataract surgery compared to the control group [37]. Uyama et al. also demonstrated positive outcomes in patients with type 2 UES, but not with type 3. They reported similar clinical and histological characteristics between types 1 and 2, as well as a positive response to decompressive scleral surgery, supporting the idea that these two types are within the same clinical spectrum and that scleral abnormalities are the responsible factor for UE in these patients, but not in type 3 UES [4].

As mentioned previously, partial or full-thickness sclerectomies were the most frequently performed procedures within the included studies, associated or not with sclerotomies and sclerostomies. Following them in frequency are isolated sclerotomies and sclerostomies. The less frequently published technique was VVD. Although it was the first published procedure by Brockhurst in 1980 [12], that has also defended its use later in 1990 [25], many authors reported this technique as difficult to perform and not showing better results when compared to sclerectomies and sclerotomies, applied together or not [3, 13, 14]. As mentioned before, different names are used for the surgical techniques among the authors. We used the names reported by the authors and attempted to focus on providing, when available, the following details regarding the procedures: removal of sclera, perforation of choroid, open or sutured sclerotomies or scleral flaps and exposure of choroid.

Furthermore, several authors referred the importance of early and correct diagnosis of UES, either idiopathic or associated with nanophthalmos, and its precocious treatment with decompressive scleral surgeries, mainly to avoid long-term RD with damage to the RPE that can prevent better outcomes postoperatively, such as improvements in VA. UES should be suspected in the presence of serous retinal detachment with shifting and high protein content of the subretinal fluid and normal IOP [3, 11]. The absence of clinical improvement with CCT and immunosuppression should also point out to this diagnosis, as it is not associated with inflammation, unlike many other diagnoses of exclusion. Finally, excluding rhegmatogenous retinal detachment and uveal melanoma is paramount since they are likely to be sight or life-threatening, respectively. Özdek et al. reported a nanophthalmic eye that had recurrence of macular detachment after undergoing sclerectomies with sclerotomies with MMC application and the authors assumed that the limited improvement was a result of retinal damage [36]. As mentioned above, Mansour et al. [34] performed partial thickness sclerectomies in five nanophthalmic eyes, associated with SRF drainage in 60%, and demonstrated a very significant improvement in VA. They hypothesized that this change in VA might have been due to a short distance between the choroid and detached retina, a highly oxygenated swollen choroid and a decreased retinal metabolism in the context of UE that prevented hypoxic damage of the retina.

In addition, Yalvac et al. defended a more anterior location of sclerectomies, 3 mm posterior to the limbus, unlike the one proposed by Brockhurst [12] for VVD, affirming it comprises lower risks of rupture and incarceration of the vitreous and retina due to a thicker uveal layer in the anterior part of the eye [42].

Cases of non-surgical management of UE have also been published. Sharma et al. reported 15 cases of UES in the UK over the course of 2 years, 7 of which were treated conservatively. Three of these did systemic therapy with steroids, two with topical steroids, one underwent laser therapy and another was maintained under observation. The median VA of these eyes remained unchanged at the last follow-up [2]. Desai et al. also reported 8 eyes with UES, 3 of which nanophthalmic, kept under observation and 14 eyes, 8 of which nanophthalmic, that were treated conservatively with steroids (topical, oral and periocular in some cases). The mean VA of both groups remained unchanged during follow-up and 10 of the 12 eyes with RD in the medical treatment group showed reattachment without surgery [26]. In fact, these results support the beforementioned idea that conservative treatment, namely with CCT, is not effective in patients with UES, unlike with other diseases such as uveitis. UES does not seem to be linked to ocular inflammation, which prevents any benefit that could arise from immunosuppressive treatment. The low efficacy of CCT in these patients could also be an important diagnostic clue of UES, as it could rule out many inflammatory diseases that are often clinically confused with it and that would have a positive response to this treatment.

It is important to be aware of possible publication bias favoring positive results, especially considering that most published articles in this topic are case reports or case series with few patients. Moreover, there are other limitations that can bias the conclusions taken in this review. Firstly, the fact that both nanophthalmia and UES are rare disorders, which reflects in studies with very few patients and makes it difficult to undertake RCTs. Secondly, the lack of a control group in most publications, either in the cases where decompressive surgeries are performed as prophylaxis for UES together with cataract or glaucoma surgeries, or in the cases where decompressive surgeries are performed for treatment of UES, in this case making it difficult to understand whether the positive outcomes are a result of the surgery or simply the natural course of the disease. Most published studies also have short follow-up times which can condition the assessment of late-stage recurrences or complications. In addition, some articles do not provide a detailed explanation of the executed surgeries making it harder to interpret and compare the obtained results between different studies and different surgeries. We contacted some authors asking for more details regarding the performed procedures but received an answer from only Rajendrababu et al. [38]. Moreover, in some articles, especially retrospective studies, some information about the patients is missing or is incomplete because it was not collected for this purpose. This can, naturally, complicate and bias the outcome interpretation. Additionally, the diagnostic criteria used for the classification of nanophthalmia is not clearly stated in many studies and, among the references that report them, it varies considerably. This compromises, once again, the comparison between studies and patients. The most frequently considered parameters for the diagnosis were a scleral thickness bigger than 1.7 mm and an AL small than 20.5 mm. However, some authors consider different cut-offs for the AL, such as 21 mm and 20 mm, and other characteristics, such as hyperopia and shallow anterior chambers. For instance, Wu et al. considered eyes with AL smaller than 21 mm, scleral thickness bigger than 1.7 mm, corneal diameter smaller than 11 mm, crowded AC and high hyperopia as nanophthalmic, whereas Yalvac et al., considered all these features, but an AL smaller than 20.5 mm as well as a high lens:eyeball ratio [40, 42].

In conclusion, this review confirms that sclerectomies, alone or combined with other techniques, are effective for treating UES and RD, particularly in patients with nanophthalmos and UES types 1 and 2. VA improvement appears to depend more on the duration of detachment and photoreceptor damage than on reattachment itself. In the future, bigger studies and RCTs with longer follow-up times are needed. Normalization of diagnostic criteria for nanophthalmia and UES would also allow more accurate comparisons and interpretation of obtained results across different studies.

## Supplementary Information

Below is the link to the electronic supplementary material.

Supplementary file1 (PDF 63 KB)

Supplementary file2 (PDF 90 KB)
