# Supplementary material for: Sclerectomies in nanophthalmos and idiopathic uveal effusion syndrome: a systematic review
Source: Graefes Arch Clin Exp Ophthalmol. 2025 Jul 15;263(10):2709–22. doi: 10.1007/s00417-025-06908-4 (PMC12583281; doi:10.1007/s00417-025-06908-4)
Supplement: Supplementary file 1 — Supplementary file1 (PDF 63 KB) [file 417_2025_6908_MOESM1_ESM.pdf]

Sclerectomies in nanophthalmos and idiopathic uveal effusion syndrome: a systematic review

Graefe's Archive for Clinical and Experimental Ophthalmology

Leonor Braga de Sousa[1], João Barbosa Breda[2,3,4]

[1] Faculty of Medicine of the University of Porto, Porto, Portugal
[2] RISE-Health, Department of Surgery and Physiology, Faculty of Medicine of the University of Porto, Porto, Portugal
[3] Department of Opththalmology, Centro Hospitalar e Universitário São João, Porto, Portugal
[4] Research Group Ophthalmology, Department of Neurosciences, KULeuven, Leuven, Belgium

Corresponding author: Leonor Braga de Sousa
leonor.sousa007@gmail.com

DATABASE	SEARCH QUERY	DATE OF SEARCH	NUMBER OF RESULTS
**PUBMED**	(sclerotom*[tw] OR sclerectom*[tw] OR "Sclerostomy"[Mesh] OR "Scleroplasty"[Mesh] OR “vortex vein decompression” [tw]) AND (nanophthalm*[tw] OR "posterior microphthalm*"[tw] OR "Microphthalmos"[Mesh] OR "Nanophthalmos 1" [Supplementary Concept] OR "Nanophthalmos 2" [Supplementary Concept] OR "Nanophthalmos 3" [Supplementary Concept] OR "Uveal Effusion Syndrome"[Mesh] OR "Uveal Diseases"[Mesh])	08/09/2024	278
**SCOPUS**	(TITLE-ABS-KEY (sclerotom* OR "sclerostomy" OR sclerectom* OR "scleroplasty" OR “vortex vein decompression”) AND TITLE-ABS-KEY (nanophthalm* OR microphthalm* OR "uveal effusion syndrome" OR "uveal diseases" OR "posterior microphthalmos"))	08/09/2024	183
**WEB OF SCIENCE**	Searched in “all fields”:<br>(sclerotom* OR “sclerostomy” OR sclerectom* OR "scleroplasty" OR “vortex vein decompression”) AND (nanophthalm* OR microphthalm* OR “uveal effusion syndrome” OR “uveal disease*” OR "posterior microphthalmos")	08/09/2024	75

Online Resource 1- Search queries.
