# Supplementary material for: Sclerectomies in nanophthalmos and idiopathic uveal effusion syndrome: a systematic review
Source: Graefes Arch Clin Exp Ophthalmol. 2025 Jul 15;263(10):2709–22. doi: 10.1007/s00417-025-06908-4 (PMC12583281; doi:10.1007/s00417-025-06908-4)
Supplement: Supplementary file 2 — Supplementary file2 (PDF 90 KB) [file 417_2025_6908_MOESM2_ESM.pdf]

Sclerectomies in nanophthalmos and idiopathic uveal effusion syndrome: a systematic review

Graefe's Archive for Clinical and Experimental Ophthalmology

Leonor Braga de Sousa1, João Barbosa Breda 2,3,4

1 Faculty of Medicine of the University of Porto, Porto, Portugal
2 RISE-Health, Department of Surgery and Physiology, Faculty of Medicine of the University of Porto, Porto, Portugal
3 Department of Opththalmology, Centro Hospitalar e Universitário São João, Porto, Portugal
4 Research Group Ophthalmology, Department of Neurosciences, KULeuven, Leuven, Belgium

Corresponding author: Leonor Braga de Sousa
leonor.sousa007@gmail.com

**Author and Year**	**D1**	**D2**	**D3**	**D4**	**D5**	**Overall**
Rajendrababu (2017)	+	+	+	!	+	!

\+ Low risk

! Some concerns

\- High risk

Domains:
D1: Bias arising from randomization process.
D2: Bias due to deviation from intended intervention.
D3: Bias due to missing outcome data.
D4: Bias in measurement of the outcome.
D5: Bias in selection of the reported result.

Online resource 2.1 - Assessment of Risk of Bias - Randomized Control Trial [24]**.**

AUTHOR AND YEAR	D1	D2	D3	D4	D5	D6	D7	D8	D9	D10
**BROCKHURST (1980)**	Y	U	U	N	N	Y	Y	Y	N	NA
**BROCKHURST (1990)**	N	U	U	U	U	Y	Y	Y	N	NA
**CASSWELL (1987)**	Y	U	U	N	N	Y	N	Y	N	NA
**DESAI (2022)**	Y	Y	Y	Y	Y	Y	N	U	U	NA
**FAULBORN (1999)**	Y	Y	Y	U	U	Y	Y	Y	N	NA
**GHAZI (2013)**	Y	Y	Y	Y	Y	Y	Y	Y	N	NA
**GUO (2019)**	Y	Y	Y	N	N	Y	Y	Y	N	NA
**JIN (1990)**	U	U	U	Y	U	Y	N	N	U	NA
**JOHNSON (1990)**	Y	U	Y	U	Y	Y	Y	Y	U	NA
**KONG (2013)**	N	U	U	N	N	Y	Y	Y	N	NA
**LIU (2018)**	Y	Y	Y	Y	Y	Y	Y	Y	N	NA
**MAGGIO (2016)**	Y	Y	Y	U	U	Y	Y	Y	N	NA
**MANSOUR (2023)**	Y	Y	Y	U	U	Y	Y	Y	N	NA
**MANSOUR (2019)**	Y	Y	Y	Y	Y	Y	Y	Y	U	NA
**ÖZDEK (2022)**	N	U	U	Y	U	Y	Y	Y	N	NA
**OZGONUL (2017)**	Y	U	U	U	U	Y	Y	Y	N	NA
**RAJENDRABABU (2020)**	Y	Y	Y	Y	Y	Y	Y	Y	U	NA
**SHAH (2016)**	Y	Y	Y	U	Y	Y	Y	U	N	NA
**SHARMA (2020)**	Y	U	U	U	U	Y	Y	Y	U	NA
**SHEN (2022)**	N	Y	U	U	U	Y	Y	Y	U	NA
**UYAMA (2000)**	Y	Y	Y	U	U	Y	Y	Y	N	NA
**WENG (1995)**	N	Y	Y	U	U	Y	Y	Y	N	NA
**WU (2004)**	Y	Y	Y	Y	Y	Y	Y	Y	N	NA
**YALVAC (2008)**	Y	Y	Y	U	U	Y	Y	Y	U	NA
**YEPEZ (2015)**	N	Y	Y	Y	U	Y	Y	Y	U	NA

Online resource 2.2 - Assessment of Risk of Bias - Case series. Y- yes; N- no; U- unclear; NA- not applicable

AUTHOR AND YEAR	D1	D2	D3	D4	D5	D6	D7	D8	D9	D10	D11
**FAN (2023)**	Y	Y	Y	N	NA	Y	Y	Y	Y	NA	Y
**ZHOU (2022)**	U	Y	Y	U	NA	Y	Y	Y	Y	NA	Y

Online resource 2.3 - Assessment of Risk of Bias - Cohort Studies. Y- yes; N- no; U- unclear; NA- not applicable
